# A rainwater control optimization design approach for airports based on a self-organizing feature map neural network model

**DOI:** 10.1371/journal.pone.0227901

**Published:** 2020-01-21

**Authors:** Dongwei Qiu, Hao Xu, Dean Luo, Qing Ye, Shaofu Li, Tong Wang, Keliang Ding

**Affiliations:** 1 School of Geomatics and Urban Spatial Informatics, Beijing University of Civil Engineering and Architecture, Beijing, P.R. China; 2 School of Humanity and Law, Beijing University of Civil Engineering and Architecture, Beijing, P.R. China; Hellenic Agricultural Organization - Demeter, GREECE

## Abstract

To address the problems of high overflow rate of pipe network inspection well and low drainage efficiency, a rainwater control optimization design approach based on a self-organizing feature map neural network model (SOFM) was proposed in this paper. These problems are caused by low precision parameter design in various rainwater control measures such as the diameter of the rainwater pipe network and the green roof area ratio. This system is to be combined with the newly built rainwater pipe control optimization design project of China International Airport in Daxing District of Beijing, China. Through the optimization adjustment of the pipe network parameters such as the diameter of the rainwater pipe network, the slope of the pipeline, and the green infrastructure (GI) parameters such as the sinking green area and the green roof area, reasonable control of airport rainfall and the construction of sustainable drainage systems can be achieved. This research indicates that compared with the result of the drainage design under the initial value of the parameter, the green roof model and the conceptual model of the mesoscale sustainable drainage system, in the case of a hundred-year torrential rainstorm, the overflow rate of pipe network inspection wells has reduced by 36% to 67.5%, the efficiency of drainage has increased by 26.3% to 61.7%, which achieves the requirements for reasonable control of airport rainwater and building a sponge airport and a sustainable drainage system.

## 1 Introduction

As an important functional area of the city, this type of airport has the characteristics of a wide area, complex land and high flood control standards. Therefore, the precisely rainwater control is a necessary condition to ensure the safe operation of the airport. Wang Shuang ling, Zhang Wan shun, and Chen Fa jin established a combined model for analyzing and simulating the drainage capacity of coastal nuclear power plants during extreme rainfall. Their design uses the predictive correction calculation method to calculate the node return flow and simulates and analyzes the flooding depth of different design rainstorm regression periods through the combined model. Through scientific analysis, this model provides a certain decision-making basis for rainwater control and control during flood disasters and provides certain security for regional sustainable development [1]. HR Wallingford successfully built a hydraulic model of the Drogheda basin using Info Works CS software, thus effectively simulating the operation of the drainage system, optimizing the layout of the pipe network, and managing the rainwater properly [2]. Manocha, Nishtha introduced an approach for automatically developing dynamic adaptation approaches using multi-objective genetic algorithms for storm water control in tropical urban catchments in Singapore [3]. Yazdi, J proposed a Monte Carlo simulation (MCS)-based probabilistic method and applied it to the western district of Tehran to improve the drainage capacity of urban drainage systems. This method is combined with the SWMM simulation model and the evolutionary search algorithm to find the best recovery measures in the blocking scenario [4]. Deitch, Matthew J, Feiler, Shane T used a cumulative effect model based on spatial GIS to simulate rainfall with a 1–5 year repeat period. They also proposed rainwater control in the Perdido River area of Florida, USA using green infrastructure and rainwater harvesting technology. Rainwater discharges in all study areas have been reduced by more than 20% [5].Mora-Melia Daniel, Lopez-Aburto Carlos S, Ballesteros-Perez Pablo et al., using the Rainwater Control Model (SWMM) software for hydrological modeling, considered rainfall runoff in different spatial regions, using different areas of green roofs controlling rainfall runoff; this method provided a useful solution to combat flooding in the Chilean region [6]. Yazdi, J proposed an elastic-based repair method for improving urban drainage systems and applied it to the Rainwater Drainage System (TSDS) in western Tehran, which integrates a multi-objective evolutionary algorithm (MOEA) and an EPA-SWMM simulation model; this was done to find cost-effective recovery measures during structural failures of key elements in the network [7]. Ashley Richard, Gersonius Berry, Digman Christopher, et al. used Green Infrastructure (GI) for the effective control of rainwater control, which has been applied to storm water control approaches throughout Europe [8]. Zheng Peng, Ke Jinyan et al. used a PCSWMM model to simulate the rainwater pipe network in the region of a high-density residential community in China [9]. Zischg Jonatan, Rogers Briony, Gunn Alexander, Rauch Wolfgang, and Sitzenfrei Robert proposed a new approach to explore the future development of urban drainage systems, emphasizing the adoption and implementation of sustainable “natural-based” storm water control strategies for establishing a multifunctional urban rainwater system. The model successfully was applied to rainwater control in the Swedish city of Kiruna [10]. Haghighatafshar Salar, Jansen Jes la Cour, Aspegren Henrik et al. introduced the concept of a mesoscale sustainable drainage system based on observed rainfall-runoff responses from two catchments in sustainable drainage systems and a pipeline catchments model. By decomposing the catchment into a group of independent small catchments, as the depth of rainfall increases, these small catchments begin to interconnect to achieve effective drainage [11].

Most scholar’s rainwater control method is to optimize the drainage network layout[12] or use the green infrastructure. The current rainwater control is mainly dependent on the gray infrastructure (such as large diameter rainwater pipe network and rainwater storage pool). Although these methods can control the rainwater to a certain extent, but in the face of heavy rainfall (take the rain on July 12, 1956 as an example), the phenomena of pipe network inspection well with high overflow rate and low drainage efficiency are caused by the low precision parameter design in various rainwater management measures (such as the diameter of the rainwater pipe network and the green roof area ratio), which affects the safe operation of the airport.

In order to better address the problem of high overflow rate and low drainage efficiency of airport pipe network inspection wells and establish a green, sustainable sponge airport, a rainwater control optimization design approach based on a self-organizing feature map neural network model (SOFM) was proposed in this paper. The SOFM model is an artificial neural network based on the principle of a competition mechanism. Through the competition between neurons, the effect of “near excitation and far suppression” of the brain nervous system occurs [13]. The SOFM model is characterized by the winner priority. Guo, Jingqiu, Liu, Yangzexi et al used the SOFM model to extract potential features of driving behavior and estimate driving risk [14]. Yan Meng-ge, Dong Xiao-zhou et al used the SOFM model combined with correlation to identify and classify the LIBS spectra of natural geological samples [15]. Wu, Ming-Chang, Hong, Jing-Shan et al used the SOFM model to analyze and forecast typhoon rainfall in Taiwan [16]. The SOFM model has become one of the most popular neural network algorithms in the data processing and analysis for various engineering purposes. The SOFM model input layer contains multiple neurons. The rainwater control design of the airport is also composed of several parameters. Multiple parameters are used as input neurons, and the winner is given priority as the criterion. Through the continuous competition between neurons, the optimal setting process of rainwater control design parameters is realized.

The study carried out hydrological space analysis based on the digital elevation model (DEM) of Beijing Daxing International Airport and simulated rainfall runoff drainage under different parameters to optimize the parameters of the construction region drainage network and the green infrastructure.

A rainwater control optimization design approach based on a self-organizing feature map neural network model (SOFM) has achieved the best design of various parameters of rainwater control in construction area. Compared with the result of the drainage design under the initial value of the parameter, the green roof model and the conceptual model of the mesoscale sustainable drainage system, in the case of a hundred-year torrential rainstorm (take the flood disaster on July 12, 1956, as an example), the efficiency of drainage has increased by 26.3% to 61.7%, the RMSE of overflow has decreased by 31% to 52.2%, the drainage efficiency of the pipe network has effectively improved, thereby achieving a better control of rainwater, effectively avoiding the occurrence of flood disasters.

## 2 Materials and methods

The optimization design of rainwater control parameters based on SOFM model is based on the storm intensity formula [17] and the airport construction requirements to determine the initial values of the rainwater pipe network design parameters and the initial values of the green infrastructure parameters to reduce the peak rainfall. The SOFM model data set was established based on the determined design parameters of the rainwater network, the design parameters of the green infrastructure and the large amount of rainfall data in the area. The parameters in the data set were used as the parameters input of the storm flood model (SWMM) [18] model and the rainfall runoff drainage simulation of the area was carried out. Optimize and adjust the parameter values of the various rainwater control measures and their weight ratio according to the overflow rate of the pipe network inspection well. The adjusted parameter values are again applied to the rainfall runoff drainage simulation. Finally, the qualified parameter values and their weight values are competitively selected by the SOFM model, and the optimal parameter values of the rainwater control parameter design can be obtained, thereby achieving the purpose of optimal design.

The specific process is as follows:

Step one: Refer to the topographical features of the airport construction area to establish a digital elevation model for the airport area. The digital elevation model is filled in to generate an innocent digital elevation model.

Step two: Through the hydrological analysis and spatial analysis of the innocent digital elevation model, the topographic parameters such as the surface water flow direction and the cumulative amount of the confluence in the construction area are determined. The division of the drainage area of the construction area is carried out in combination with the terrain parameters of the construction area and the actual land use planning.

Step three: According to the storm intensity formula, the storm flood model and the airport construction requirements, the initial values of the rainwater pipe network parameters and the initial values of the green infrastructure parameters were determined. The data set of the SOFM model is established using the initial values of each parameter and the rainfall data of the construction area over the years.

Step four: The parameters in the SOFM model are input into the storm flood model (SWMM) to simulate the rainfall runoff drainage. By using the pipe network to check the overflow ratio of the well as a basis for judging, it is determined whether the initial value of the parameter meets the design requirements.

Step five: When the test results are judged to be unqualified, the initial values and weights of the parameters are adjusted using the SOFM model, until the test results are acceptable.

**Fig 1:**
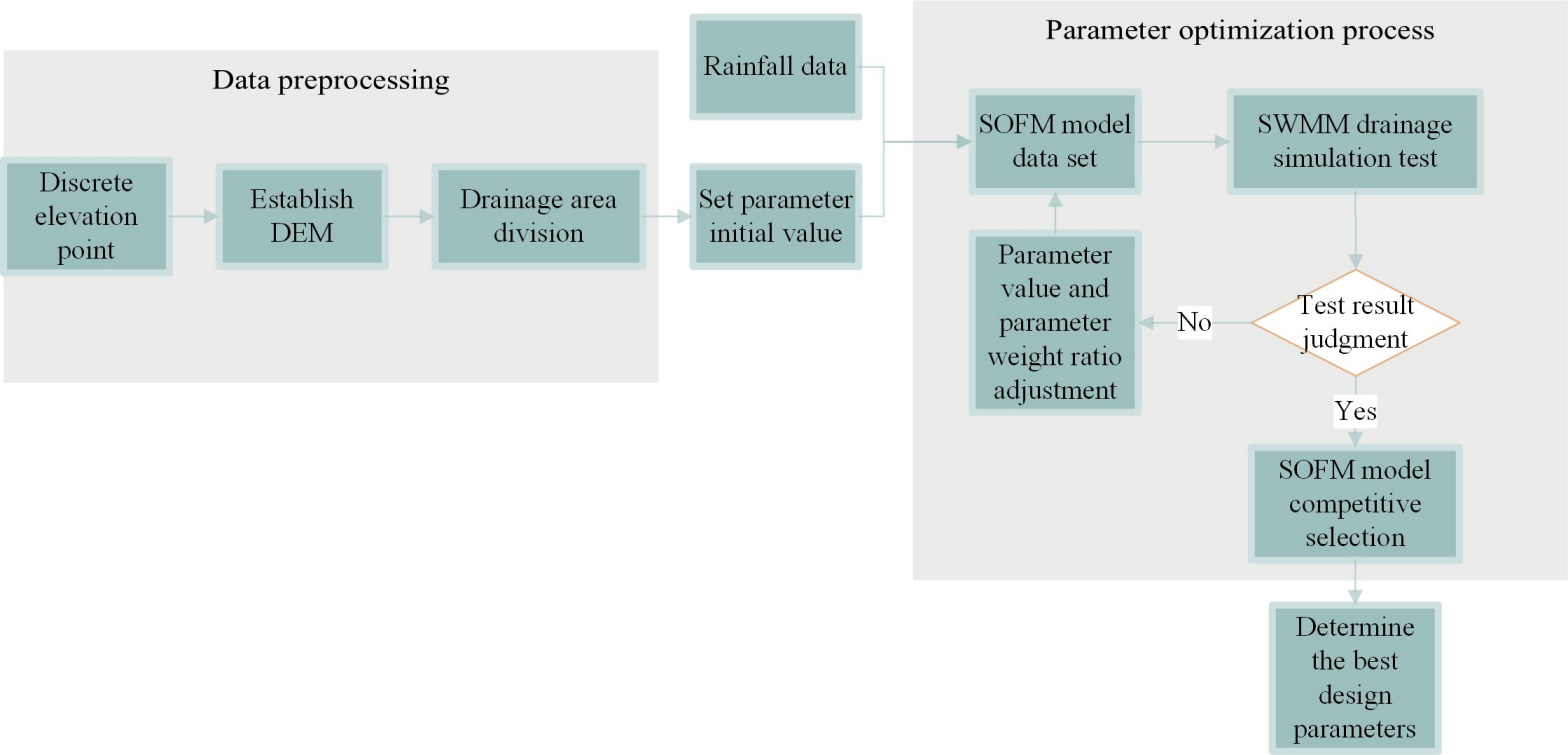
Flow chart of rainwater control optimization design.

Step six: Using the SOFM model, the qualified parameters of various control measures are competitively selected to determine the optimal design parameters of the rainwater control model. The design process of rainwater control optimization is shown in Fig 1.

### 2.1 Data preprocessing

**Fig 2:**
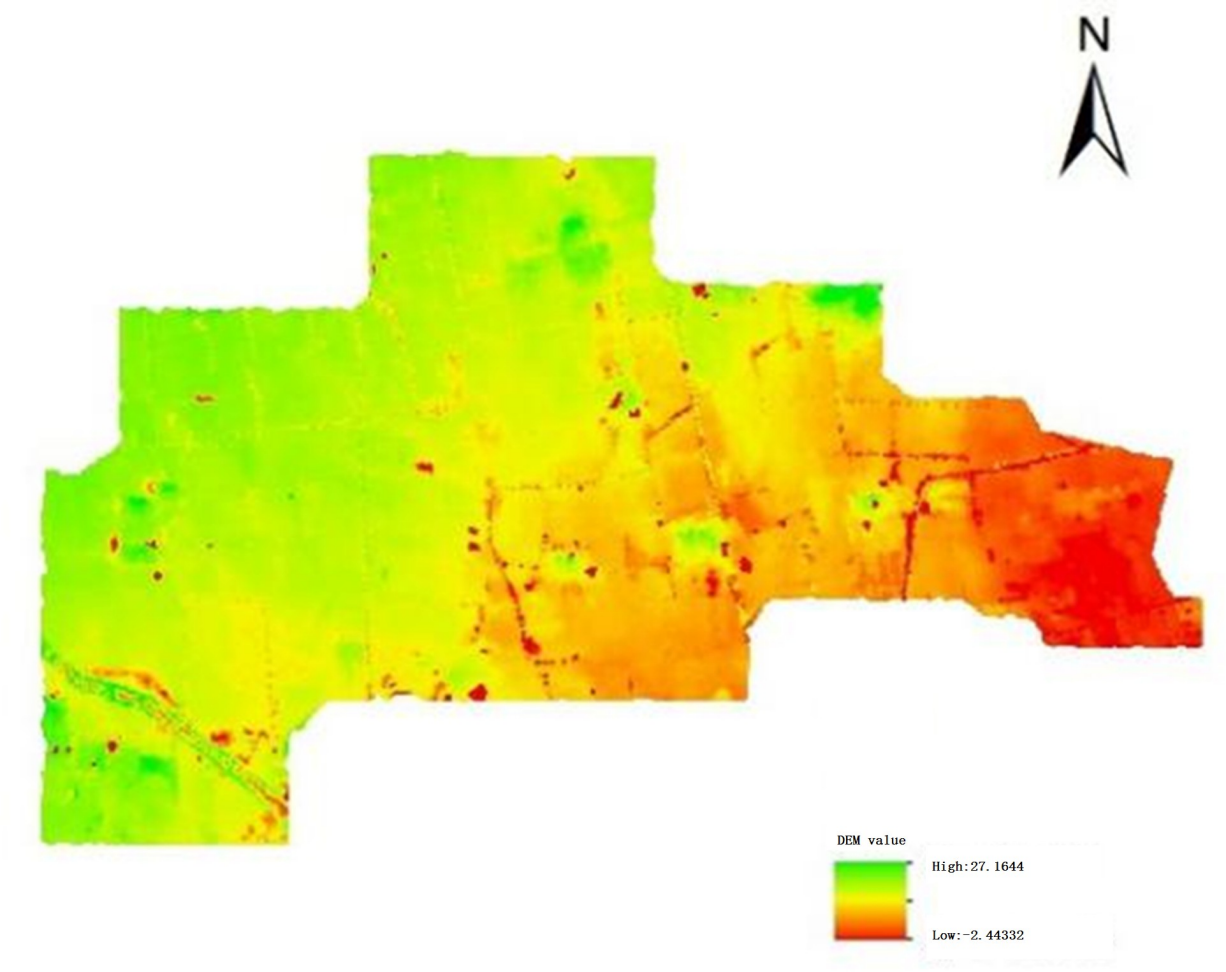
Innocent DEM.

The data preprocessing work mainly includes the establishment of digital elevation model, filling of digital elevation model, hydrological space analysis, and division of drainage area. The digital elevation model (DEM) is built using the discrete elevation points of the airport construction area, and the improved PF algorithm [19] is used to level the depression in the original DEM. The digital elevation model (DEM) after filling is the basis for the establishment of the entire drainage network model. The Innocent DEM is shown in Fig 2.

Surface runoff always flows from high to low ground and finally flows to a water outlet. Therefore, to scientifically and accurately identify the watershed boundary, the flow direction of water flow in each grid unit must be determined first. According to different algorithm principles, the algorithms for extracting the flow data and the accumulation of the flow are divided into two types: a single flow algorithm and multi-flow algorithm[20]. Considering the size of the data and the quality requirements of the design model for the data analysis results, the maximum slope single flow algorithm [21–22] is used to extract the flow direction and the aggregate cumulative data in the innocent DEM.

The division of the drainage area is usually carried out by dividing the watershed and the catchment network. The design model of this study locates all connected grid cell groups belonging to the same drainage basin by analyzing the input surface flow to the raster data and then creates a basin through locating the pour point at the edge and identifying the confluence area at each pour point.

### 2.2 Set parameter initial value

**Fig 3:**
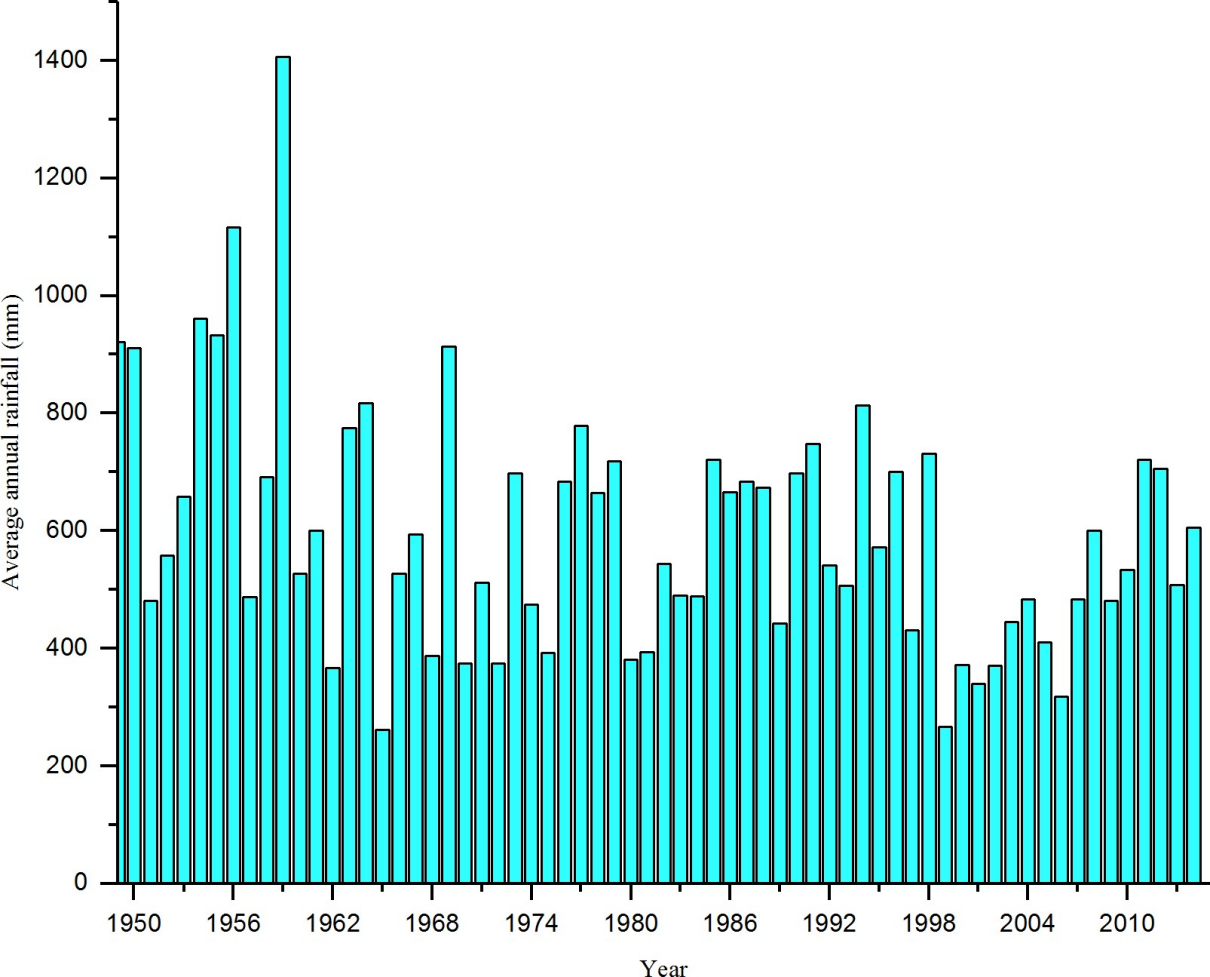
Statistical graph of annual average rainfall in Beijing from 1949 to 2014.

The initial value setting of the rainwater control model parameters mainly includes the setting of the initial values of various parameters of the rainwater pipe network and the initial values of various parameters of the green infrastructure. This study uses a large amount of rainfall data from 1949 to 2014 as a reference (as shown in Fig 3), combined with the previously defined drainage area and refers to the storm intensity formula and the 1D/2D flood drainage runoff simulation [23] to set the initial values of the parameters of the rainwater network. The SWMM model [24] was used to set the design return period P of the rainwater pipe network in the construction area. The selection of the return period P of the rainwater pipe network needs to consider the topographical characteristics and construction properties of the construction area. For areas with serious consequences from flooding, the value of P ranges from 5 to 10 years, and each drainage basin can choose a different design return period.

The storm intensity formula reflects the relationship between design rainstorm intensity q, rainfall duration t, and rainwater return period P. This is an important design basis for the rainwater pipe network, and its calculation model is shown in formula (1):

$$q=\frac{167A_{1}*\left(1+c*\mathrm{lg}p\right)}{{\left(t+b\right)}^{n}}$$ (1)

where q(L/s/ha) is the design storm intensity, p(year) is the design rainfall return period, t (min) is rainfall time, and *A*_1_, c, and b are local parameters, and they are determined according to statistical methods.

The formula for calculating the design discharge Q of rainwater pipe network is shown in Formula (2).

Q=Ψ*q*A (2)

where Q (L/s) is the rainwater design discharge, A (*hm*^2^) is the drainage area, and Ψ is the runoff coefficient.

The formula for calculating *q*_0_ of runoff per unit area is shown in Formula (3):

$$q_{0}=q*\;\Psi$$ (3)

The velocity formula is shown in formula (4):

$$V=R^{2/3}*I^{1/2}/n$$ (4)

where V (*m*/*s*) is the velocity, n is the roughness coefficient, R (m) is the hydraulic radius, and I is the hydraulic gradient.

The formula of effective section area of flow is shown in formula (5):

A=Q/V (5)

where Q (*m*^3^/*s*) is the discharge, A (*m*^2^) is the effective section area of flow, and V (*m*/*s*) is the velocity.

The roughness coefficients of concrete prefabricated pipes or polyethylene pipes are 0.013 and 0.009, respectively, and the effective velocity range of the two drainage pipes is 0.75–4.0 *m*/*s*.

Rainwater control is an urgent issue facing public spaces at this stage, as more and more impervious surfaces destroy natural hydrology. Water resource managers are increasingly turning to more environmentally friendly ways to collect rainwater, which is called Green Infrastructure (GI) [25]. The green infrastructure [25, 26, 27] mainly consists of permeable floor coverings, sunken green spaces, and green roofs.

The primary role of the green infrastructure is to reduce the runoff into the stormwater pipeline by rainwater absorption during rainfall. Sinking green spaces and green roofs increase water storage capacity, thereby reducing surface runoff. The main contribution of sinking green spaces is increased retention and evapotranspiration, while green roofs allow for more retention through the water in their matrix. According to the literature [26], effective green infrastructure in the face of heavy rainfall can reduce rainwater runoff by 23%-42%. Green roofs can control rainfall runoff at the source, while sinking green spaces and permeable floor coverings can control rainfall runoff during the process. According to the airport construction requirements, the sinking green space ratio is not less than 50%, the ground permeable pavement rate is not less than 15%, and the green roof ratio is not less than 30%.

The sinking green rate calculation model is shown in formula (6):

$$g=\frac{g_{1}}{g_{2}}*\;100\%$$ (6)

where, *g*_1_(*m*^2^) is the area of sinking green space in the region, *g*_2_(*m*^2^) is the total green area in the region

The ground permeable pavement rate calculation model is shown in formula (7):

$$k=\frac{k_{1}}{k_{2}}*\;100\%$$ (7)

where, *k*_1_(*m*^2^) is the area of ground permeable pavement area in this region, *k*_2_(*m*^2^) is the area of hardened ground area in this region.

The green roof ratio calculation model is shown in formula (8):

$$R=\frac{R_{1}}{R_{2}}*\;100\%$$ (8)

where, *R*_1_(*m*^2^) is the area of design green roof, *R*_2_(*m*^2^) is the total area of planned building roof.

### 2.3 Design parameter optimization

The current rainwater control s mainly dependent on the gray infrastructure (such as large diameter rainwater pipe network and rainwater storage pool). In the case of reducing the peak rainfall, the phenomenon of pipe network inspection well with high overflow rate and low drainage efficiency is caused by low precision parameter design in various rainwater control measures (such as the diameter of the rainwater pipe network and the green roof area ratio). In order to address this problem better, the rainwater control optimization method based on SOFM model is introduced here, combined with the local rainfall data, the rainwater control design model is optimized to minimize the overflow rate of the rainwater network inspection well. The corresponding parameters are the best design standards for the rainwater control facilities.

#### 2.3.1 Optimization objective function

The relationship between the design parameters of the airport rainwater control facility and rainfall is shown in formula (9):

$$p\leq \frac{\pi r^{2}}{4}*V+\frac{\pi }{n}*{\left(\frac{r^{8}}{1024}\right)}^{\frac{1}{3}}*I^{\frac{1}{2}}+\sum_{i=1}^{Ni}c*k_{wc}*16\%*h_{c}+\sum_{i=1}^{Ni}d*k_{wd}*58\%+\sum_{i=1}^{Ni}z*k_{wz}*12\%*h_{z}$$ (9)

Where, *p* (mm) is the rainfall; *r* (mm) is the rainwater pipe diameter parameter; *n* is the roughness coefficient; V (*m*/*s*) is the velocity; I is the hydraulic gradient; *c*(*m*^2^) is the surface permeable pavement area; *k*_*wc*_(*cm*/*s*) is the permeability coefficient of permeable pavement; *h*_*c*_(cm) is the permeable paving thickness; *d* (*m*^2^) is the sinking green area; *k*_*wd*_(*cm*/*s*) is the permeability coefficient of sinking green area; z(*m*^2^) is the area of the green roof; *k*_*wz*_(*cm*/*s*) is the permeability coefficient of green roof; *h*_*z*_(cm) is the green roof thickness; The permeability coefficients of permeable paving, sinking green area and green roofs are determined by their materials.

The optimization objective function model of airport rainwater control is shown in formula (10):

$$\left\{ \begin{array}{c} S=\frac{\sum_{i=1}^{Ni}y}{\sum_{i=1}^{Ni}Y}=\mathrm{Min},(0\leq S\leq 10\%)\\ RMSE=\sqrt{\frac{\sum {\left(S_{SOFM}-S_{Other}\right)}^{2}}{N}}=Min,(RMSE\geq 0)\\ NSEC=1-\frac{\sum {\left(s_{\mathrm{SOFM}}-s_{\mathrm{Other}}\right)}^{2}}{\sum {\left(s_{\mathrm{SOFM}}-{\bar{s}}_{\mathrm{Other}}\right)}^{2}}=Max,(0\leq NSEC\leq 1) \end{array}\right.$$ (10)

Where, s(%) is the check well overflow ratio; RMSE is the pipe network inspection well overflow flow root mean square error; NSEC is the efficiency coefficient; Ni is the watershed area number; y is the number of overflow inspection wells, and Y is the total number of inspection wells in the pipe network; *S*_*SOFM*_ is the overflow ratio of node inspection wells optimized by the SOFM simulation for design parameters of the pipeline network. *S*_*Other*_ is the overflow ratio of pipe network node inspection wells predicted by other models with the same pipe network parameters. N is the number of pipe network design parameter optimization times. ${\bar{s}}_{Other}$ is the mean value of the overflow ratio of node inspection wells predicted by other models.

When the objective function requirement shown in formula (10) is satisfied, the optimal parameter combination U of the corresponding airport rainwater pipe control can meet the optimization design requirements. The optimal design parameter combination U for airport rainwater control includes pipe diameter parameters *r*, rainfall *p*, slope coefficient of pipeline *h*, Manning roughness coefficient of pipeline *n*, permeable pavement area *c*, sunken green area *d* and green roof area z. The optimal design parameter combination U is as shown in formula (11):

U={r,p,h,n,c,d,z} (11)

Where, *r*(mm) is the rainwater pipe diameter parameter; *p*(mm) is the rainfall; *h*(%) is the slope coefficient of the pipeline; *n* is the pipeline Manning roughness coefficient, *c* (*m*^2^) is the permeable pavement area, and *d* (*m*^2^) is the sinking green area. z(*m*^2^) is the area of the green roof.

At the same time, other optimization design targets for airport rainwater control are when the total overflow rate of the pipe network inspection well is less than 10%, the efficiency coefficient at more than 85%, and the sinking green area ratio at no less than 50%. The pavement permeable rate is not less than 15%, the ratio of green roof is not less than 30%, and the regional overflow warning value is 45 *m*^3^/*s*. The pipe network layout is as small as possible, with a small burial depth and low cost.

#### 2.3.2 Parameter optimization process

In the data pre-processing section, the airport construction area has been divided into several drainage areas. The divided drainage area is used as the experimental environment, and the SOFM model data set is established by collecting the rainfall data of the area over the years and the initial values of various control parameters. A single rainfall runoff drainage simulation was performed using the parameters in the SOFM model data set as parameter inputs to the SWMM model. The initial value of each parameter weight is set according to its importance in airport rainwater control. In a single training simulation, rainfall is a decisive factor in the parameter adjustment of rainwater control facilities, so the weight of rainfall is always 1. The initial values of the weights of the six parameter values except for the rainfall are equally distributed in proportion.

In each process of rainfall runoff simulation, the overflow rate, root mean square error (RMSE), efficiency coefficient (NSEC) and total runoff control ratio of pipeline inspection wells were used as the main criterion. Less than 10% of the inspection well overflow rate in each area is considered qualified. The root mean square error (RMSE), efficiency coefficient (NSEC), and total runoff control ratio are used as the compare reference for comparison of rainfall runoff drainage simulation under different parameters. According to the evaluation criteria, the control model parameters that meet the design conditions are retained, and the unqualified parameters are eliminated.

**Fig 4:**
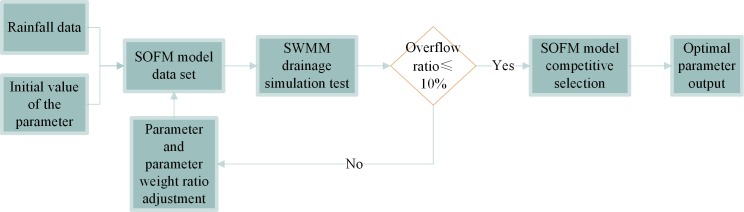
SOFM neural network model diagram.

The schematic diagram of the SOFM model is shown in Fig 4. The input of the control model parameters is shown in formula (12):

$$net_{i}\left(t\right)=w_{1}*r\left(t\right)+w_{2}*p\left(t\right)+w_{3}*h\left(t\right)+w_{4}*n\left(t\right)+w_{5}*c\left(t\right)+w_{6}*d\left(t\right)+w_{7}*z\left(t\right)$$ (12)

where *i* is the order number of the input layer; *t*(min) is the rainfall time, and *w*_1_ is the current pipe diameter parameter weight, *w*_1_∈[0,1]. *r*(*t*)(mm) is the rainwater pipe diameter parameter, *w*_2_ is the weight of the current rainfall, and the value range is *w*_2_∈[0,1]. *p*(*t*) (mm) is the rainfall, *w*_3_ is the weight of the slope coefficient of the current pipeline, and the value range is *w*_3_∈[0,1]. *h*(*t*) is the slope coefficient of the pipeline; *w*_4_ is the current pipeline’s Manning roughness coefficient weight. The value range is *w*_4_∈[0,1]. *n* is the pipeline’s Manning roughness coefficient, and *w*_5_ is the weight of the ground permeable pavement area. The value range is *w*_5_∈[0,1]. *c*(*t*)(*m*^2^) is the surface permeable pavement area. *w*_6_ is the weight of the sunken green area, and the value range is *w*_6_∈[0,1]. *d*(*t*) (*m*^2^) is the sinking green area. *w*_7_ is the weight of the green roof area, and the value range is *w*_7_∈[0,1]. z(*t*)(*m*^2^) is the area of the green roof.

The weight adjustment range of the six parameter values except rainfall is determined according to the adjustable range of its corresponding parameters. When the adjustment range of the parameter value is small, the weight adjustment range corresponding to the parameter is relatively large; when the parameter adjustment range is large, the weight adjustment range corresponding to the parameter is relatively small. For example, the adjustment range of the pipeline Manning roughness coefficient is relatively small, so the corresponding weight adjustment should be appropriately expanded; the adjustment range of the rainwater pipe diameter and the green roof area is relatively large, so the corresponding weight adjustment amount should be reduced appropriately.

The seven parameter weight adjustment models are shown in formula (13):

$$w_{i+1}=w_{i}\pm s*a_{n}$$ (13)

Where, *w*_*i*+1_ is parameter weight value after the adjustment of each parameter; *w*_*i*_ is the previous parameter weight value of each parameter; s is overflow ratio of inspection wells in the area under current parameters; *a*_*n*_ is the system weight adjustment proportional coefficient corresponding to each parameter, the specific value is determined by statistical analysis; n is serial number code of each parameter, 1–7 refers to rainwater pipe diameter, rainfall, pipeline slope coefficient, pipeline Manning roughness coefficient, ground permeable pavement area, sinking green area, and green roof area.

According to the overflow rate of each overflow, the size of the seven weight indicators and the above parameters are adjusted separately. When the overflow rate is high, the pipe diameter and the slope of the pipe should be increased, the pipe with low Manning roughness coefficient should be selected, and the permeable ground pavement area and sunken green area should be expanded to alleviate the high overflow rate of the drainage area.

After many adjustments of various parameters, the control parameters corresponding to the overflow ratio S less than 10% are competitively selected. Calculated the distance between all input values and their corresponding weight values, the function model of parameter competition selected is shown in the formula (14):

$$\begin{array}{l} d\left(r,p,b,n,c,d,z,w\right)={\left\|r-w_{1}\right\|}^{2}+{\left\|p-w_{2}\right\|}^{2}+{\left\|h-w_{3}\right\|}^{2}+{\left\|n-w_{4}\right\|}^{2}+{\left\|c-w_{5}\right\|}^{2}+{\left\|d-w_{6}\right\|}^{2}\\ =\sum_{i=1}^{n}\left({\left(r-w_{1}\right)}^{2}+{\left(p-w_{2}\right)}^{2}+{\left(h-w_{3}\right)}^{2}+{\left(n-w_{4}\right)}^{2}+{\left(c-w_{5}\right)}^{2}+{\left(d-w_{6}\right)}^{2}+{\left(z-w_{7}\right)}^{2}\right) \end{array}$$ (14)

According to the principle of the winner priority in the self-organizing feature mapping neural network (SOFM), the shortest neuron in all neurons must be found and defined as the winner, as shown in formula (15):

$$winner=\arg\;\min\{d\left(r,p,h,n,c,d,z,w_{i}\right)\}$$ (15)

As shown in formula (16), only the output result of the winning neuron is 1, that is, the corresponding parameter combination U reaches the optimal design requirement; Otherwise, the results of other neurons are 0, that is the corresponding parameter combination U does not meet the optimal design requirements.

$$f(U)=\{\begin{array}{c} 1,winner\\ 0,otherwise \end{array}$$ (16)

At this time, the output result of the corresponding parameter combination U is the optimal design parameter of the airport rainwater control model, and the best design parameter results are output.

When the pipe network inspection well overflow ratio S meets the requirements, the root mean square error RMSE and the efficiency coefficient NSEC of the well overflow flow can be checked through the pipe network to evaluate the accuracy of the control model. When the root mean square error is smaller and the efficiency coefficient is larger, the control effect of the model is better.

The root mean square error evaluation formula is shown in formula (17), and the efficiency coefficient evaluation formula is shown in formula (18):

$$RMSE=\sqrt{\frac{\sum {\left(S_{SOFM}-S_{Other}\right)}^{2}}{N}}$$ (17)

$$NSEC=1-\frac{\sum {\left(s_{SOFM}-s_{Other}\right)}^{2}}{\sum {\left(s_{SOFM}-{\bar{s}}_{Other}\right)}^{2}}$$ (18)

Where *S*_*SOFM*_ is the overflow ratio of node inspection wells optimized by the SOFM simulation for design parameters of the pipeline network. *S*_*Other*_ is the overflow ratio of pipe network node inspection wells predicted by other models with the same pipe network parameters. N is the number of pipe network design parameter optimization times. ${\bar{s}}_{Other}$ is the mean value of the overflow ratio of node inspection wells predicted by other models.

## 3 Case study

### 3.1 Study region

The International Airport in Daxing District of Beijing in China is a large international transportation hub. It will greatly ease the traffic pressure in the capital region. The construction of the new airport must meet the requirements of green, environmental protection and sustainable development. A reliable rainwater control design is the necessary guarantee for the safe operation of the new airport.

The International Airport is located in Yufa Town, Daxing District, Beijing; it is 4.3 km away from the Beijing-Kowloon Railway to the west, approximately 1 km from the embankment on the north bank of the Yongding River in the south, and covers an area of approximately 29*km*^2^. The new airport is bounded by the Xintian River in the north and the Yongding River in the south, as a part of the greater Yongding River system. The Xintian River runs through the central part of the construction area from the northwest to the southeast (the river trend has changed). The airport rainwater control design project is constructed according to the requirements of the sponge airport.

### 3.2 Airport drainage area division

**Fig 5:**
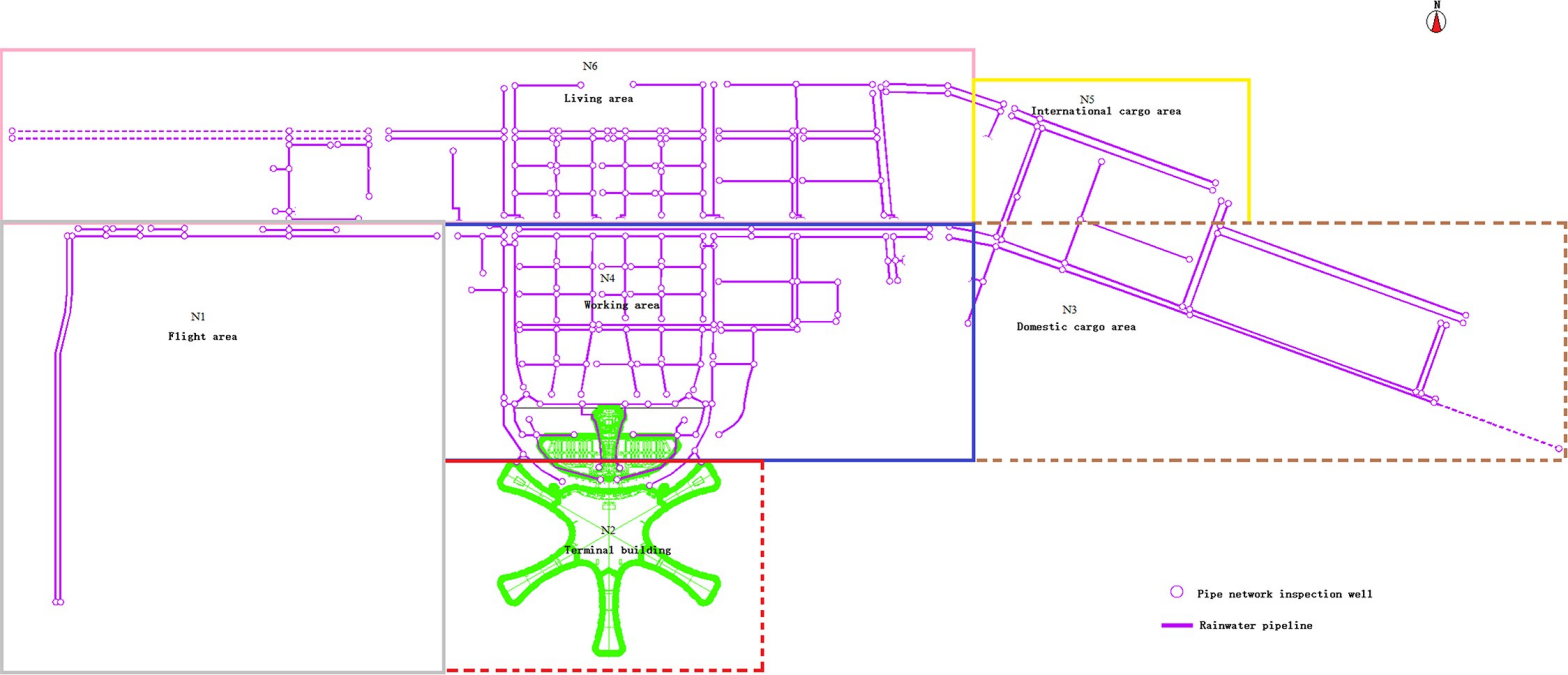
Airport rainwater drainage area map.

The data preprocessing work of the airport construction area is carried out. Based on the discrete elevation points of the construction area, the digital elevation model (DEM) of the International Airport is established and the depression filling is carried out. The hydrological analysis is made by using the spatial analysis method, and the preliminary division of the catchment area is carried out by using the result of the flow direction extraction. After considering the topographical factors of the airport and the actual land use planning of the airport, the design divides the airport into six rainwater drainage areas, which are represented by N1, N2, N3, N4, N5, and N6. Area N1 is the flight area of the airport, area N2 is the airport terminal area, area N3 is the domestic cargo area, area N4 is the airport work area, area N5 is the international cargo area, and area N6 is the airport living area. The final result of the division of drainage areas is shown in Fig 5.

### 3.3 Results

#### 3.3.1 The result of parameter initial value

The initial value of the pipe parameters is the basic data for the optimization design of the pipeline parameters. The value mainly includes parameters such as the drainage design flow, pipe diameter, embedding slope and flow velocity of each drainage area. According to the formula of storm intensity (1) and the formula of rainwater pipe network design flow (2), the discharge design flow value Q of each drainage area is calculated, as shown in Table 1.

**Table 1:** Drainage design flow value table for each drainage area.

Area number	Drainage area (*hm*^2^)	runoff coefficient	Drainage design flow value(*m*^3^/*s*)
N1	605	0.70	75.0
N2	258	0.68	41.0
N3	306	0.55	21.0
N4	246	0.68	30.3
N5	415	0.80	86.0
N6	175	0.60	43.0

The initial value data of the pipe network parameters is calculated by the calculation formulas (1)–(5), and the calculation results are shown in Table 2.

**Table 2:** Pipe network parameter initial value data.

Area number	Pipe diameter(mm)	Design flow rate(*m*/*s*)	Slope(‰)	Maximum depth(m)	The depth ratio(%)
N1	3800	2.75	4.3	3.6	80
N2	3400	3.0	6.0	4.4	95
N3	3300	2.7	4.0	2.8	76
N4	3500	2.8	4.5	3.8	68
N5	3200	3.8	9.8	5.6	40
N6	3600	2.5	3.8	3.3	97

The layout of the rainwater pipe network in the six drainage areas is mainly determined by the location of the building and the direction of the existing road, and the open channel or hidden pipe is chosen according to the actual situation of the region. The layout of rainwater drainage outlets is determined according to the slope direction of the ground and the catchment area of each pipe section. In the design of rainwater systems, drainage outlets are often set up at or near the outflow point. The catching tool of the tipping point in a hydrological analysis module can be used to capture the outlet of the catchment area. Combined with the capture results of the outlet and the current situation of the new Tian tang River, the total drainage outlet is finally located on the east side of the site. According to the results of rainwater drainage zoning and slope analysis, the rainwater pipes are laid along the road according to the gravity flow discharge method, and drainage open channels are planned to be arranged in the north, east and south of the airport.

The comprehensive runoff coefficient of the rainwater drainage area is mainly affected by the topography, average slope, vegetation and soil characteristics of the catchment area, which is usually determined according to the properties of surface materials in each drainage area. The rainwater catchment area of the land-side area and the runoff coefficient of a five-year rainfall are shown in Table 3.

**Table 3:** Landside rainwater drainage area table.

Area number	Drainage area (*hm*^2^)	runoff coefficient
N1	605	0.70
N2	258	0.68
N3	306	0.55
N4	246	0.68
N5	415	0.80
N6	175	0.60

#### 3.3.2 The result of parameter optimization

The optimal design of rainwater control in Beijing’s Daxing International Airport is mainly from the airport's green infrastructure and rainwater pipe network. Therefore, the final optimization design results are mainly divided into the optimal design result of green infrastructure and the optimal design result of the rainwater pipe network. The optimal design of the green infrastructure mainly includes the permeable pavement rate, the sinking green space ratio and the green roof ratio. The optimal design results of the green infrastructure are shown in Table 4.

**Table 4:** Optimal design results of green infrastructure in each district.

Area number	Permeable paving rate(%)		Sink green ratio (%)	Green roof ratio(%)
N1		58	52	65
N2		62	56	63
N3		65	53	72
N4		55	55	62
N5		60	51	68
N6		66	58	75

The optimal design result of various parameters of the rainwater pipe network is to construct a first-stage rainwater regulating pool[28] downstream of the designed rainwater pipelines of the three rainwater drainage areas of N1, N3 and N5 in this design. The N1 area pipeline adopts a concrete prefabricated pipeline, and the downstream is connected to the N1 first stage rainwater regulation tank and is pumped up to the drainage open channel after the first stage rainwater pumping station. The N3 area pipeline adopts a concrete prefabricated pipeline, while the downstream is connected to the N3 first stage rainwater regulation tank and is pumped up to the artificial lake after the first stage rainwater pumping station. The rainwater pipeline in area N2 is made of polyethylene pipe, and the downstream flow reaches the downstream portion of the rainwater in the N3 working area and is connected to the N3 first-grade rainwater regulating pool. The N5 area pipeline adopts a concrete prefabricated pipeline, and the downstream is connected to the N5 first-grade rainwater regulating pool and is pumped up to the artificial lake after the first-stage rainwater pumping station. The pipeline in area N6 is made of polyethylene pipe, and the downstream is directly connected to the drainage open channel. The optimal design results of the detailed parameters of the rainwater pipe network are shown in Table 5, the optimal design results of the weight values of various parameters of the rainwater control model are shown in Table 6.

**Table 5:** Optimal design results of various parameters of rainwater pipe network.

Area number	Pipe diameter(mm)	Design flow rate(*m*/*s*)	Slope(‰)	Maximum depth(m)	Minimum depth(m)
N1	4000	2.62	4.2	3.8	1.2
N2	3600	2.70	4.0	4.1	1.3
N3	3300	2.62	2.8	3.4	1.0
N4	3600	2.75	3.2	3.6	1.5
N5	3000	2.58	3.6	4.8	1.5
N6	3800	2.65	3.6	3.7	1.0

**Table 6:** Weight values of various parameters of rainwater control model.

Area number	r	p	h	n	c	z
N1	0.21	1	0.10	0.18	0.20	0.12
N2	0.19	1	0.12	0.16	0.21	0.13
N3	0.20	1	0.12	0.18	0.20	0.13
N4	0.22	1	0.13	0.17	0.19	0.11
N5	0.21	1	0.12	0.18	0.19	0.14
N6	0.20	1	0.13	0.16	0.17	0.16

## 4 Discussion

To verify the effectiveness and rationality of the SOFM model for airport rainwater control during heavy rainfall, this paper selects the result of the drainage design under the initial value of the parameter, the green roof model and the conceptual model of the mesoscale sustainable drainage system for comparison. The storm intensity formula and the storm flood model are the normative design models for various rainwater drainage designs in the world, it is the basis for setting the initial value of the pipe network. The green roof model is based on a storm flood model, and with the development of modern airports, green roofs have been widely used in various drainage projects [6]. The conceptual model of the mesoscale sustainable drainage system is to decompose the catchment into multiple independent small catchments, and the airport is also composed of six separate catchments [11]. The application scenarios of these three models are very similar to the actual situation of the airport, and these three models are also commonly used for rainwater control, so they have certain comparative value.

Rainwater control design using a SOFM model can withstand heavy hundred-year rains, the efficiency of drainage has increased by 26.3% to 61.7%, the RMSE of overflow has decreased by 31% to 52.2%,these upgrades effectively improve the drainage efficiency of the pipe network and more effectively realize the reasonable control of the rainwater, effectively avoiding the occurrence of flood disasters.

The SOFM model method is based on the parameter initial value and rainfall data as the parameter input, and simulates the regional drainage condition of N1-N6 under the different design parameters, so as to obtain the optimal design parameters of the N1-N6 regional pipe network design. Among the rainfall data in the past years, the most representative is the largest rainfall data in Beijing's history. The rainfall data is shown in Table 7.

**Table 7:** Five representative historical rainfall values.

Year	1956	1959	1969	1994	2011
Average annual rainfall(mm)	1111.5	1406	913.2	813.2	721.3

**Fig 6:**
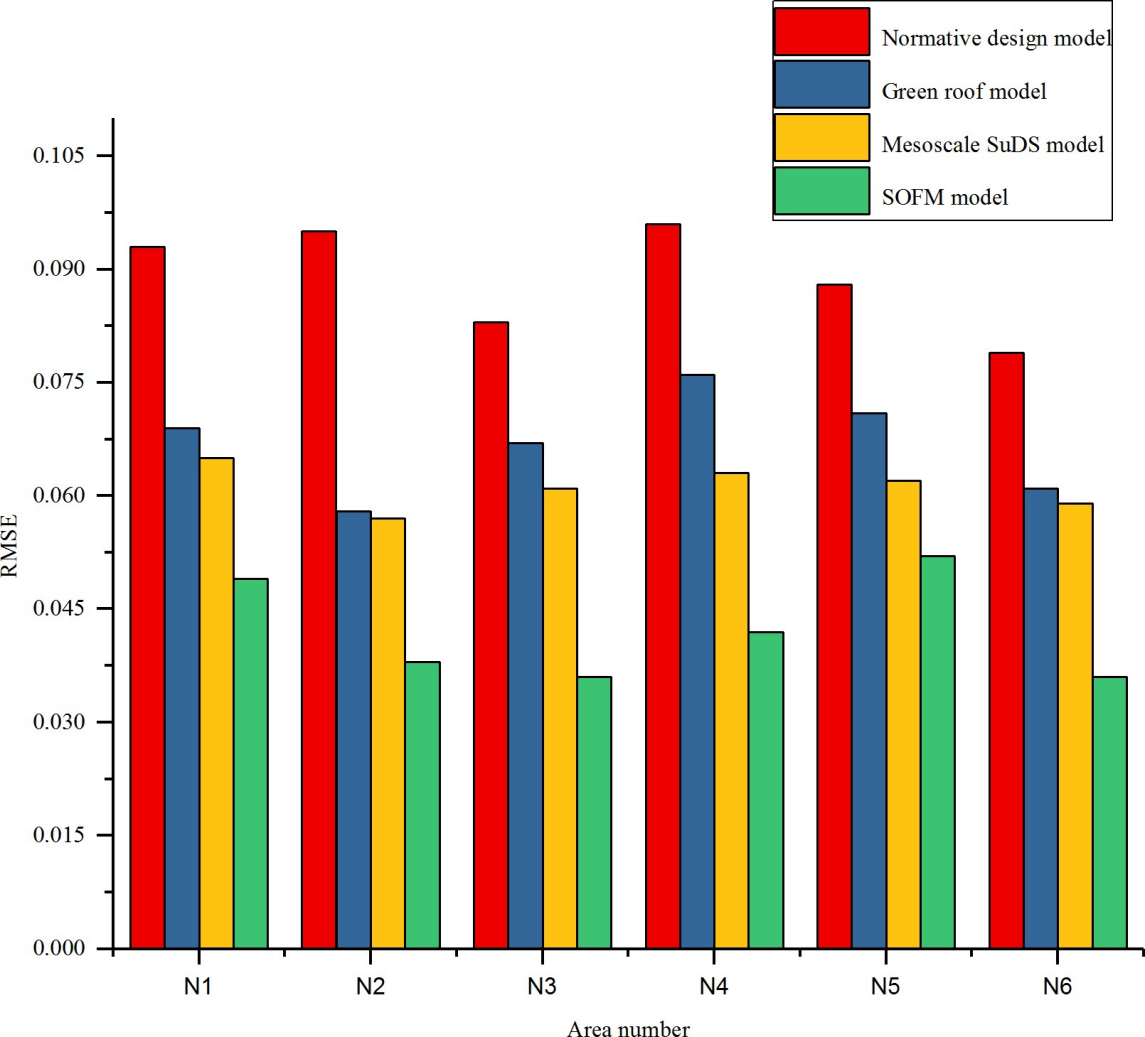
Root mean square error diagram of inspection well overflow in each area.

**Fig 7:**
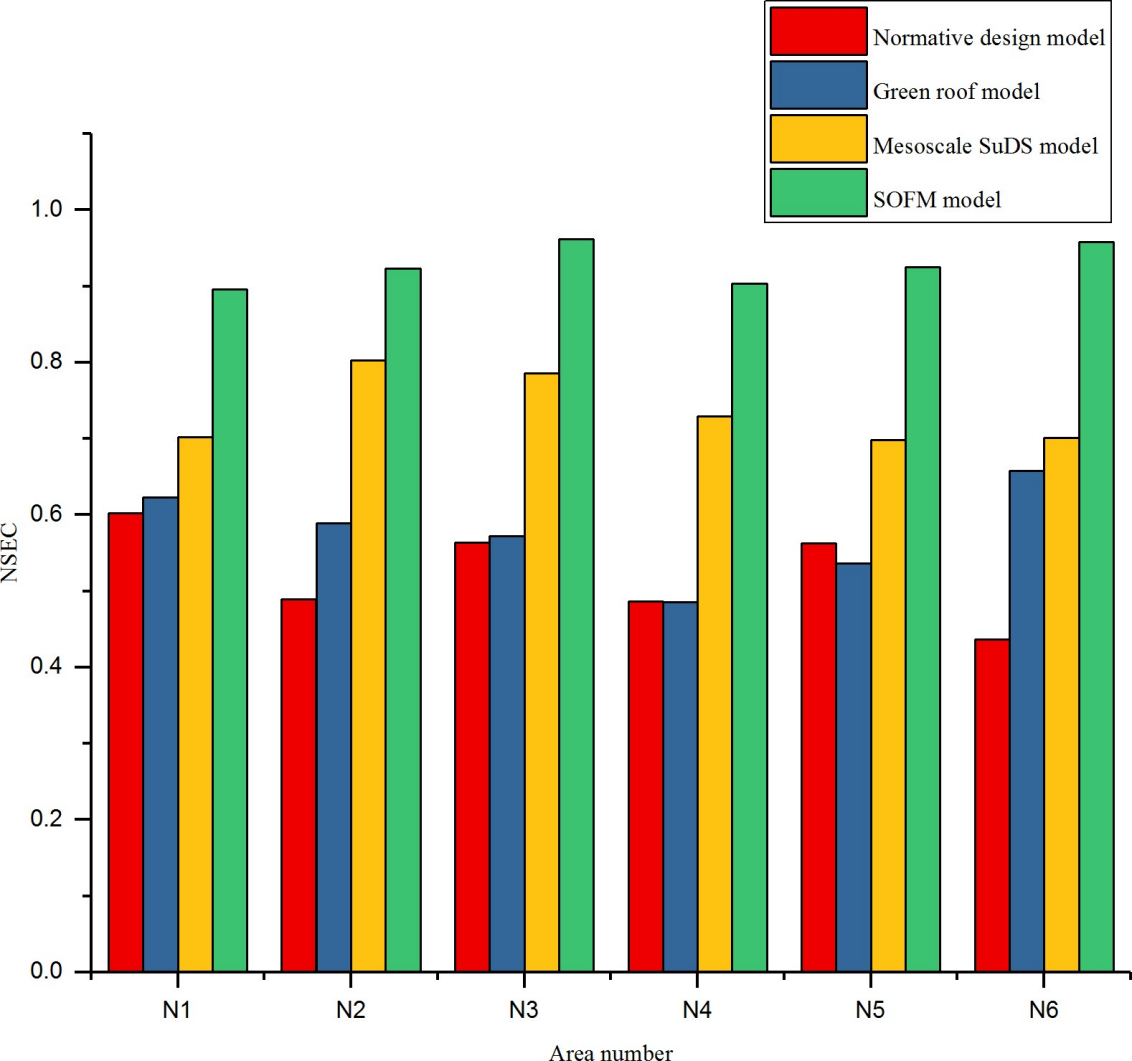
Efficiency index of inspection wells in each area.

**Fig 8:**
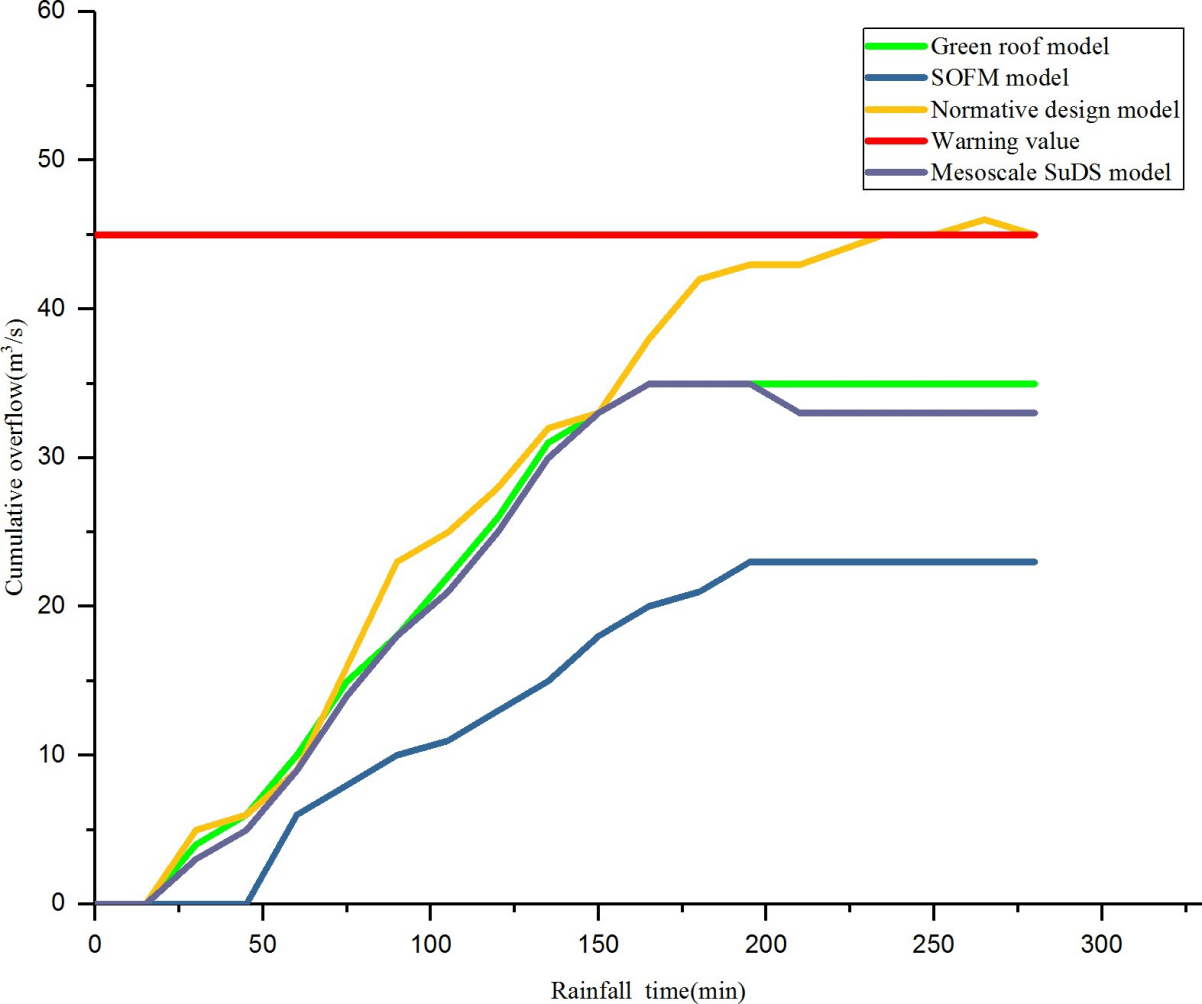
The relationship between rainfall time and cumulative overflow in area N1.

**Fig 9:**
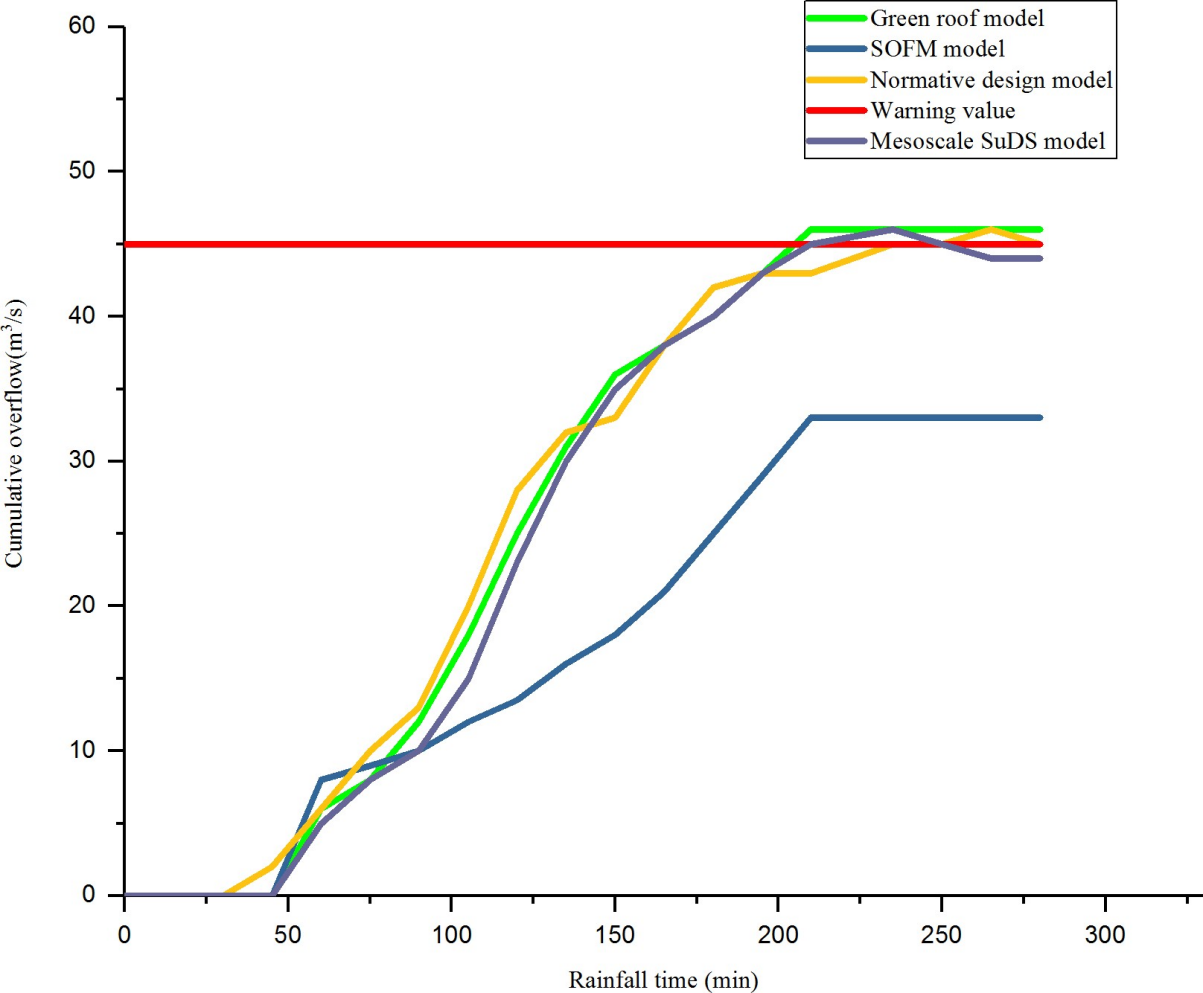
The relationship between rainfall time and cumulative overflow in area N5.

The RMSE of an overflow simulation of checking wells after 60 minutes of a rainstorm on July 12, 1956, in different regions is shown in Fig 6. The efficiency of checking wells in each region is shown in Fig 7. The relationship between the rainfall time and cumulative overflow of rainwater are shown in Fig 8 and Fig 9 (taking N1 and N5 as examples).

As shown in Fig 8 and Fig 9, compared with the result of the drainage design under the initial value of the parameter, the green roof model and the conceptual model of the mesoscale sustainable drainage system, the SOFM-based model approach considers the pipe diameter of the pipe network, the pipe embedding slope, the pipe network parameters such as pipe materials, the green roof area, the permeable pavement area, and the sinking green space; through the adjustment of the values of various parameters, the effective control of airport rainwater during heavy rainfall is realized. In the first 30 minutes of rainfall, there was no overflow in the rainwater control in the four models; the four drainage areas based on the result of the drainage design under the initial value of the parameter were first flooded at 45 minutes, 42 minutes, 46 minutes, and 43 minutes, respectively. As the rainfall time increases, the green roof model and the mesoscale sustainable drainage system begin to overflow, and the SOFM model finally overflows. In the overflow velocity, the result of the drainage design under the initial value of the parameter is larger than the green roof model result, the green roof model result is larger than the mesoscale sustainable drainage system model result, and the SOFM model has the smallest overflow velocity. After 210 minutes of rainfall, the overflow of the SOFM model began to decline first, followed by the mesoscale sustainable drainage system model, the green roof model, and the result of the drainage design under the initial value of the parameter.

**Fig 10:**
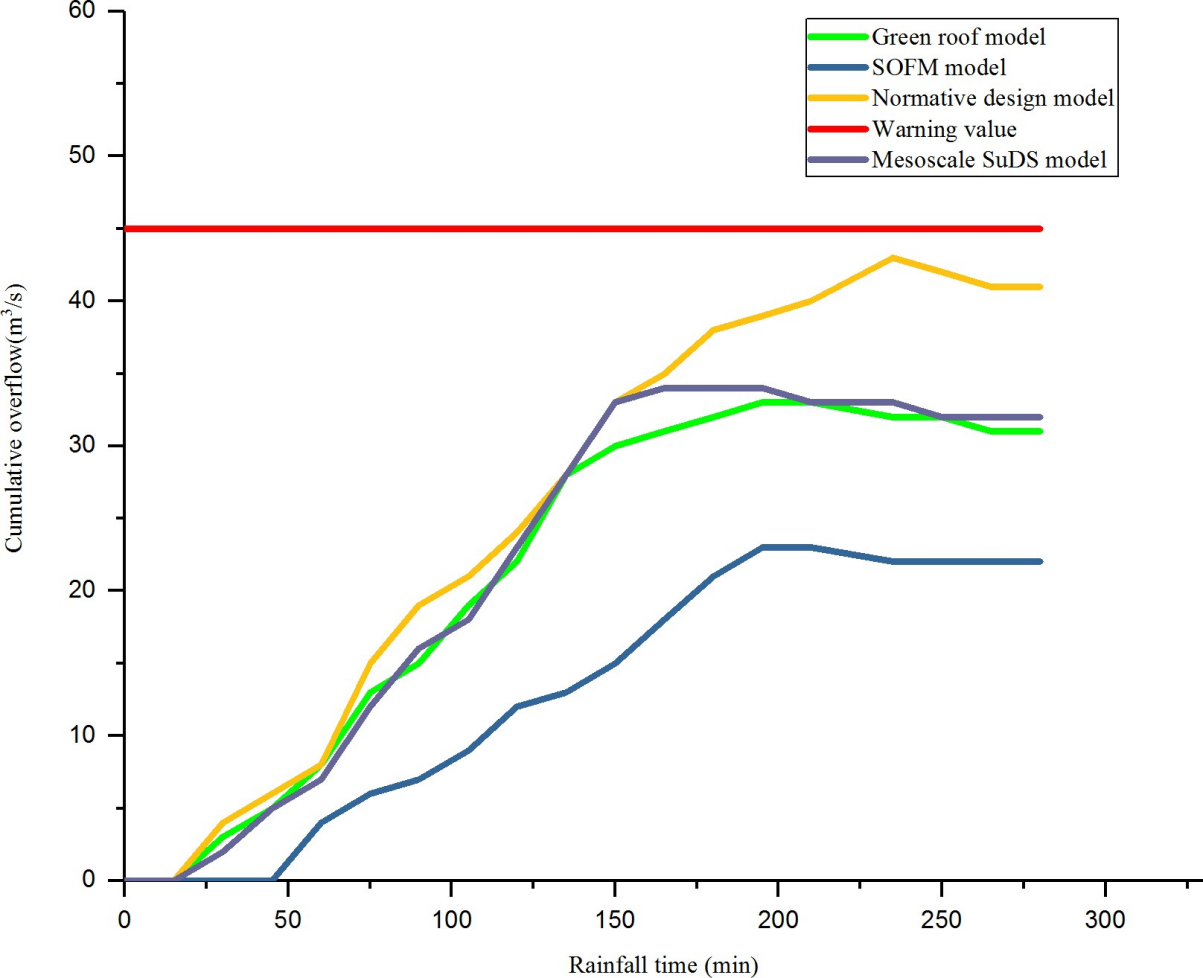
N1 (after adding regulating pool) rainfall time and cumulative overflow.

**Fig 11:**
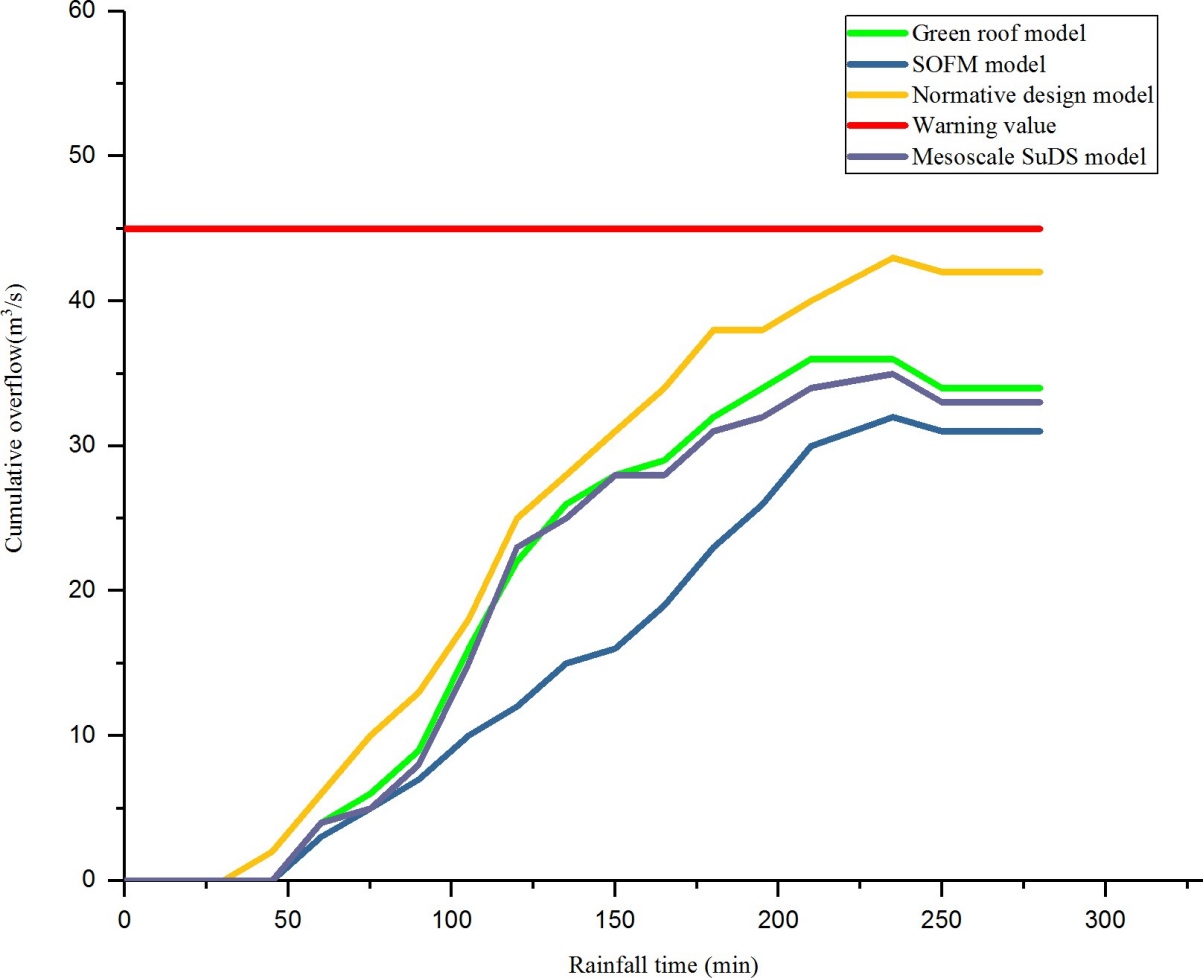
N3 (after adding regulating pool) rainfall time and cumulative overflow.

**Fig 12:**
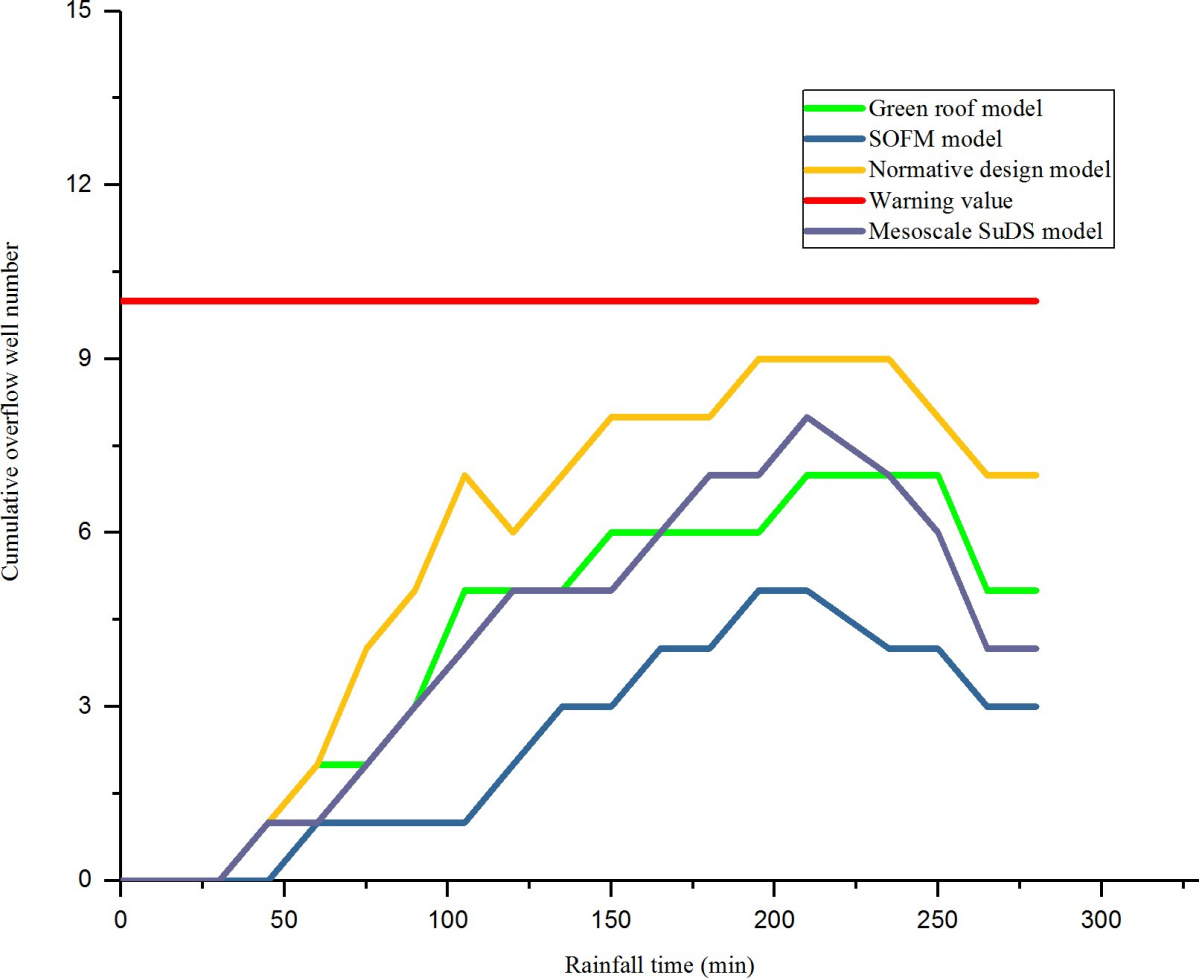
N5 (after adding regulating pool) rainfall time and cumulative overflow well number.

As shown in Fig 8 and Fig 9, for the problem of high drainage overflow rate in the N1, N3, and N5 areas. In this design, one rainwater pumping station and regulating tank are proposed in the downstream of these three areas, and the rainfall runoff drainage simulation is carried out again. The relationship between the overflow of the three areas after adjustment and the time of the rain are shown in Fig 10, Fig 11 and Fig 12.

As shown in Fig 10, Fig 11 and Fig 12, after adding rainwater pumping stations and regulating pools in areas with high overflow rate, the overflow situation of inspection wells simulated by various models improved. Compared with the scenarios that did not add rainwater regulating pools, the cumulative overflow rate of inspection wells was reduced by approximately 20%, which met the drainage requirements of constructing a sponge airport and sustainable drainage. Compared with the result of the drainage design under the initial value of the parameter, the green roof model and the conceptual model of the mesoscale sustainable drainage system, the rainwater pipe control design based on the SOFM model approach generally reduces the cumulative overflow and cumulative overflow ratio of the pipe network in the storm scenario. Compared with the result of the drainage design under the initial value of the parameter, the cumulative overflow rate of inspection wells decreased by 67.5%; compared with the green roof model, the cumulative overflow rate of inspection wells decreased by 45%; compared with conceptual models of mesoscale sustainable drainage systems, the cumulative overflow rate of inspection wells decreased by 36%.

Because Beijing Daxing International Airport is in the temperate semi-humid monsoon climate zone, the rainy season is concentrated in June to July every year, and the airport is close to the the Xintiantang River and the Yongding River, rainwater can be smoothly discharged through the rainwater pipe network and stored through the green infrastructure in the face of short-term heavy rainfall. Compared with other methods, the time delay of the overflow of the airport rainwater control based on the self-organizing feature map neural network model is delayed by 10–30 minutes, and the instantaneous cumulative overflow is also generally reduced by 17–31 *m*^3^. However, since the root mean square error of the overflow is only 0.035, when the airport is located in the tropical monsoon climate and there is no river in the surrounding area, the rainwater pipe control optimization design based on the self-organizing feature map neural network model may generate overflow flow simulation error. Inspection wells are prone to large flooding, which in turn causes flooding. Therefore, the current rainwater control optimization design has certain geographical and climatic conditions, and the root mean square error of the overflow is still at a large level.

## 5 Conclusions

To address the problems of high overflow rate of pipe network inspection well and low drainage efficiency, a rainwater control optimization design approach based on a self-organizing feature map neural network model (SOFM) was proposed in this paper. These problems are caused by low precision parameter design in various rainwater control measures such as the diameter of the rainwater pipe network and the green roof area ratio. Through the optimization adjustment of the pipe network parameters such as the diameter of the rainwater pipe network, the slope of the pipeline, and the green infrastructure (GI) parameters such as the sinking green area and the green roof area, reasonable control of airport rainfall and the construction of sustainable drainage systems can be achieved. The main conclusions are as follows:

1. Compared with the result of the drainage design under the initial value of the parameter, the green roof model and the conceptual model of the mesoscale sustainable drainage system, this model uses the self-organizing feature map neural network (SOFM) for the optimal design of rainwater control. In the case of a hundred-year torrential rainstorm, the overflow rate of pipe network inspection wells has reduced by 36% to 67.5%, the efficiency of drainage has increased by 26.3% to 61.7%.

2. The model can adjust various parameters of the rainwater network and the green infrastructure, and use these parameters as parameters for rainfall runoff drainage simulation. It can effectively improve the parameter design accuracy of airport rainwater control facilities, it has provided a better decision-making basis for airport rainwater control design and effectively avoids airport flooding.

## Supporting information

S1 File Rainfall data. (XLSX)

S2 File Rainwater pipe network design data. (XLSX)

S3 File Green infrastructure design data. (XLSX)

S4 File Fig 6 data. (XLSX)

S5 File Fig 7 data. (XLSX)

S6 File Fig 8 data. (XLSX)

S7 File Fig 9 data. (XLSX)

S8 File Fig 10 data. (XLSX)

S9 File Fig 11 data. (XLSX)

S10 File Fig 12 data. (XLSX)
